# Characteristics of resistance training-based protocols in older adults with sarcopenic obesity: a scoping review of training procedure recommendations

**DOI:** 10.3389/fnut.2023.1179832

**Published:** 2023-05-10

**Authors:** Leonardo Santos Lopes da Silva, Leonardo da Silva Gonçalves, Pedro Pugliesi Abdalla, Cícero Jonas Rodrigues Benjamim, Márcio Fernando Tasinafo, Ana Cláudia Rossini Venturini, Lucimere Bohn, Jorge Mota, Pablo Jorge Marcos-Pardo, Wolfgang Kemmler, André Pereira dos Santos, Dalmo Roberto Lopes Machado

**Affiliations:** ^1^School of Physical Education and Sport of Ribeirão Preto (EEFERP/USP), University of São Paulo, Ribeirão Preto, Brazil; ^2^Study and Research Group in Anthropometry, Training, and Sport (GEPEATE), School of Physical Education and Sport of Ribeirao Preto, University of São Paulo, Ribeirão Preto, Brazil; ^3^Department of Internal Medicine, Ribeirão Preto Medical School, University of São Paulo, Ribeirão Preto, Brazil; ^4^Faculty of Psychology, Education and Sport, Lusófona University, Porto, Portugal; ^5^Research Center in Physical Activity, Health and Leisure (CIAFEL), Faculty of Sports, University of Porto, Porto, Portugal; ^6^Faculty of Sports, University of Porto, Porto, Portugal; ^7^Neuropsychological Evaluation and Rehabilitation (CERNEP) Research Centre, Scientific Projects Organization and Research Training (SPORT) Research Group (CTS-1024), Department of Education, Faculty of Education Sciences, University of Almería, Almería, Spain; ^8^Active Aging, Exercise and Health/HEALTHY-AGE Network, Consejo Superior de Deportes, Ministry of Culture and Sport of Spain, Madrid, Spain; ^9^Institute of Medical Physics, Friedrich-Alexander-University of Erlangen-Nürnberg, Erlangen, Germany; ^10^Institute of Radiology, University Hospital Erlangen, Erlangen, Germany; ^11^Ribeirão Preto College of Nursing, University of São Paulo, Ribeirão Preto, Brazil; ^12^Escola Superior de Educação e Comunicação, Campus da Penha, University of Algarve, Faro, Portugal

**Keywords:** sarcopenia, body composition, strength training, periodization, exercise training

## Abstract

**Background:**

Sarcopenic obesity (SO) is a clinical and functional disease characterized by the coexistence of obesity and sarcopenia. Resistance training (RT) characteristics for older adults with sarcopenia or obesity are already well established in the scientific literature. Nonetheless, we still do not know how detailed the RT protocols are described for older adults with SO. Therefore, we aimed to analyze the characteristics of RT programs, including each of their variables, recommended for older adults with SO.

**Methods:**

This is a scoping review study that was conducted in accordance with the Preferred Reporting Items for Systematic Reviews and Meta-Analysis for Scoping Reviews. The search was carried out until November 2022 in PubMed/MEDLINE, EMBASE, Cochrane Library, Web of Science, Scopus, LILACS, Google Scholar, and medRxiv databases. The studies included SO diagnosis and RT as an intervention strategy. The RT variables analyzed were as follows: exercise selection, the volume of sets, the intensity of load, repetition cadence, rest interval between sets, and weekly frequency.

**Results:**

A total of 1,693 studies were identified. After applying the exclusion criteria, 15 studies were included in the final analysis. The duration of the RT intervention ranged from 8 to 24 weeks. All studies included full-body routines, with single/multi-joint exercises. Regarding the volume of sets, some studies fixed it in three sets, whereas others varied between one and three sets. The load was reported by repetition range and the weight lifted, elastic-band color/resistance, percentage of one repetition maximum, or perceived exertion scale. Repetition cadence was fixed in some studies, while it was self-selected between concentric and eccentric phases in others. The interval between sets of rest varied from 30 to 180 s. All studies reported progression overload during the interventions. Not all studies reported how the exercise selection, repetition cadence, and rest interval were made.

**Conclusion:**

The characteristics of RT protocols and their variables prescribed in the literature for older adults with SO were mapped. The lack of detail on some training variables (i.e., exercise selection, repetition cadence, and rest interval) was identified. RT protocols are heterogeneous and described only partially among studies. The recommendations for RT prescription details in older adults with SO are provided for future studies.

**Systematic review registration:**

https://osf.io/wzk3d/.

## 1. Introduction

Sarcopenic Obesity (SO) is characterized by the co-occurrence of sarcopenia and obesity in older adults (1, 2). SO is a significant health issue for older adults due to the interaction between decreased skeletal muscle mass quantity, low strength, and excessive adiposity (1, 2). This phenomenon leads to poor metabolic, cardiovascular, and functional outcomes and negatively impacts the quality of life (3–6). Declines in grip strength, gait speed, and physical performance tasks are the most evident physical and functional impairments in older adults with SO (7–9). Older adults with SO have potential consequences, such as disability, hospitalization, and increased healthcare costs, at an estimated total cost of $40.4 billion (10). Moreover, it has been proposed that SO independently predicts mortality from all causes (9). In this sense, health professionals have focused on the deleterious effects of SO (11).

Resistance training (RT) is a well-established exercise intervention that counteracts sarcopenia (12, 13) and obesity (14) in older adults. The increase in skeletal muscle mass, muscle strength, physical performance parameters (i.e., rate of force development in different tasks of daily living activities), and decrease in relative adipose tissue are the main benefits arising from RT in older adults (15). In general, RT can achieve health benefits for older adults; sessions of 8 to 12 single or multi-joint exercises are recommended, in a volume that varies from two to three sets of low/moderate intensity (8–15 maximum repetitions) (16). Older adults are told to perform each exercise with a 1 s duration for concentric muscle actions and 2 s for eccentric actions, with rest intervals of 90–180 s between sets. Regarding frequency, two–three training sessions are recommended per week (15, 17). In this way, RT recommendations for older adults with SO are supposed to be the same for sarcopenic or obese older adults. Despite this assumption, no consensus exists regarding RT characteristics for older adults with SO. There remains a gap in the literature regarding how RT is prescribed for older adults with SO.

To advance the field of knowledge about RT for older adults with SO, it is necessary to map the literature on the characteristics of RT protocols prescribed for this population. Thereby, this mapping will provide a comprehensive understanding of exercise prescription strategies, enabling exercise professionals to guide their interventions more effectively. Based on the information presented earlier, we raise the following question: What are the characteristics of RT-based protocols prescribed for older adults with SO? Therefore, this scoping review aimed to understand the characteristics of RT protocols prescribed for older adults with SO by mapping the methodological processes addressed in this field. We hypothesize that RT protocols for older adults with SO provide a detailed characterization of the variables that make up the training.

## 2. Methods

### 2.1. Protocol and registration

This is a scoping review study of the scientific literature (18) performed based on the stages proposed by Preferred Reporting Items for Systematic Reviews and Meta-Analysis (PRISMA) for Scoping Reviews (19). Our review was registered on Open Science Framework (https://osf.io/wzk3d/) (20). The Preferred Reporting Items for Systematic Reviews and Meta-Analyses extension for Scoping Reviews (PRISMA-ScR) checklist is presented in Supplementary Table 1.

Scoping reviews are more suitable for assessing and understanding the extent of knowledge in an emerging field. In addition, they can identify, map, report, and discuss the characteristics or concepts in that field (21, 22). As a precursor to a systematic review, scoping reviews aim to examine how research is conducted on a certain topic or field, to clarify whether a systematic review can be conducted to address a specific question after mapping the literature (21, 22). Therefore, scoping reviews are an important tool for researchers to gain a broad understanding of a field and identify research gaps, which can help to guide future research and improve the quality of evidence-based decision-making.

### 2.2. Review question

The review question was formulated and organized with the PCC strategy, as proposed by Peters et al. (21). Therefore, Problem: SO; Concept: RT protocols; Context: detailed RT variables according to National Strength; and Conditioning Association recommendations: exercise selection, the volume of sets, load intensity, repetition cadence, rest interval between sets, weekly frequency, and duration of RT intervention (23).

### 2.3. Eligibility criteria

This review included primary studies with clinical trials (randomized or controlled) or observational (cross-sectional or cohort) designs from peer-reviewed papers, pre-print, dissertation, thesis, and conference abstracts. The studies considered older adult participants with the diagnosis of SO (according to the criteria of each study). RT protocols featuring free weights [i.e., with or without external overload (weights, elastic band)] and machines were considered. For protocols with combined exercises (e.g., RT and endurance), only the RT information was extracted. Protocols with other types of exercises [i.e., only endurance (in treadmill or bicycle and multi-component)] were not included in the scope of this review.

### 2.4. Search strategy

The search for studies was carried out in the following databases: MEDLINE (*via* PubMed), EMBASE, Cochrane Library, Web of Science, SCOPUS, and LILACS. Furthermore, the gray literature was consulted using Google Scholar and medRxiv. The search strategy was formulated from a combination of controlled descriptor (using boolean operators “AND” or “OR”) keywords related to the topic, without applying restrictions related to language or publication periods was also applied. The search syntax was composed of the keywords as follows: “resistance training” OR “resistance exercise” OR “strength training” OR “strength exercise” OR “weight exercise” OR “weight training” OR “exercise training” AND sarcopeni^*^ AND obes^*^. The search strategy in each specific base is presented in Supplementary Table 2. We performed the searches in the databases up to 26 November 2022.

###  2.5. Study selection

The identified studies were imported into EndNote Basic to remove the duplicates and then imported into the Rayyan software. Studies without duplicates were evaluated and selected based on eligibility criteria by two independent and blinded reviewers (LS and LG) by reading the title and abstract of the studies (phase 1), followed by reading the full text of the selected studies in phase 1 (phase 2). Furthermore, the lists of references cited by selected studies in phase 2 were analyzed to identify other eligible studies to be also included in this review (snowballing method). A third reviewer (MFTJ) solved disagreements in the study selection process.

### 2.6. Data extraction

The data from selected studies were rigorously analyzed and collected from two independent and blind reviews, by filling out a characterization table in Microsoft Excel software, which contains information such as:

– Characteristics of the study: identification (citation), study design, and RT protocol (exercises selected, volume of sets, intensity of load, repetition cadence, rest interval between sets, weekly frequency, and duration of the intervention);
– Characteristics of individuals: sample size, sex, average age, and which measures they were diagnosed as sarcopenic obese.

At the end of this process, a cross-checking of all information retrieved was carried out. A third review by an experienced proofreader resolved the divergences.

### 2.7. Data analysis

A qualitative synthesis of the selected studies' data was conducted, which included a description of sample characteristics (average age and sex), the diagnostic criteria for SO, and the main characteristics of RT protocols. The main variables analyzed in the resistance programs were the exercises used in the studies, the volume of sets, the intensity of load, repetition cadence, rest interval between sets, weekly frequency, and protocol duration.

## 3. Results

**Figure 1:**
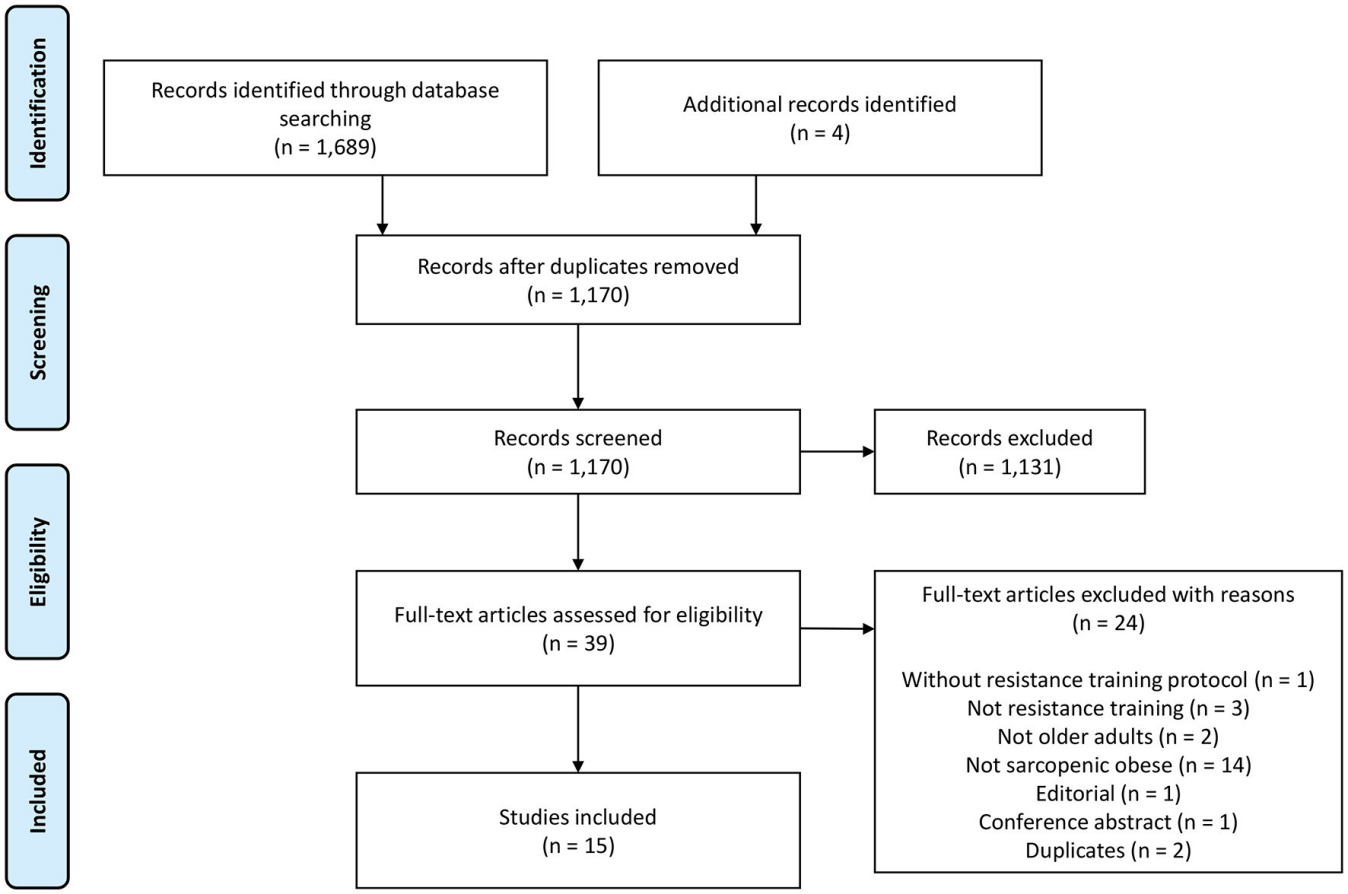
Flowchart of PRISMA for scoping reviews.

Figure 1 presents the flow diagram of the present study. Initially, 1,693 studies were identified. After removing duplicates, 1,170 titles and abstracts were screened, resulting in 39 potentially eligible full texts. A total of 15 studies (24–38) were included in this review.

A summary of the included studies is presented in Table 1. There was heterogeneity between the diagnostic criteria of SO, especially in the different assessment tools (DXA, BIA, BMI, anthropometric equations, grip strength, gait speed, and short physical performance battery). All studies performed a full-body routine, with exercises for the main muscle groups. Exercises selected, repetition cadence, and rest interval were the only variables not reported by all studies. The length of RT intervention ranged from 8 to 24 weeks. All studies reported progression in overload during the protocol development (by repetitions range and weight lifted, elastic band color, percentage of repetition maximum, or the rating perceived exertion scale).

**Table 1:** Characteristics of the included studies, considering the sample characterization and detailed resistance training protocol.

**Author**	**Sample characterization**			**Resistance training protocol**									
	**Age**	**Sex**	**Sarcopenic obesity criteria**	**Exercises**	**Volume of sets**	**Repetitions**	**Load**	**Absolute intensity**	**Repetition cadence**	**Rest interval (s)**	**Weekly frequency (% of adherence)**	**Duration (weeks)**	**There was a progressive load during the study?**
Balachandran et al. (24)	71 ± 8	F/M	M = SMI < 10.76kg/m^2^; handgrip < 30 kg; gait speed < 1 m/s; BMI >30 kg/m^2^ F = SMI < 6.76 kg/m^2^; gait speed < 1 m/s; handgrip strength < 20 kg; BMI >30 kg/m^2^ **Sarcopenia** assessed by BIA	Leg press, triceps pushdown, seated row, chest press, lat pulldown, biceps curl, leg curl, hip adduction, calf raise, shoulder press, and hip adduction	3	10-12	50-80%1RM	-	Concentric as fast as possible and eccentric in 2s.	60-120	2 (85%)	15	Yes
Kim et al. (27)	81 ± 4	F	SMI < 5,67 kg/m^2^; body fat ≥32% and grip strength < 17 kg; or body fat ≥32% and walking speed < 1 m/s **Sarcopenia** assessed by BIA	Toe lift, heel lift, knee lift, and knee extension (sitting on a chair); hip flexions, lateral leg raises, and repetitions of other exercises (standing behind the chair and holding onto the back); leg extensions, hip flexions, double arm-pulldown, biceps curl, seated row, leg press, abduction, leg extension, abdominal crunch machine	1–3	10	Adjusted by the repetitions range	-	-	-	2 (-)	12	Yes
	**Age**	**Sex**	**Sarcopenic obesity criteria**	**Exercises**	**Volume of sets**	**Repetitions**	**Load**	**Absolute intensity**	**Repetition cadence**	**Rest interval (s)**	**Weekly frequency (% of adherence)**	**Duration (weeks)**	**There was a progressive load during the study?**
Shen et al. (26)	≥ 65	F/M	M = ASM < 7.0 kg/m^2^; handgrip < 26 kg; gait speed ≤ 0.8 m/s; body fat >26.3% F= < 5.7 kg/m^2^; handgrip < 18 kg; gait speed ≤ 0.8 m/s; body fat >33.2% **Sarcopenia** assessed by BIA	Lower extremity exercise with an elastic band for stretching and squatting and upper extremity exercise with the lifting of dumbbells	1–3	6–12	65–80%1RM	-	-	-	3 (-)	8	Yes
Gadelha et al. (25)	66 ± 5	F	regression equation FFM = 13,012 + 16,737 [Stature (m)] + 0.07231 [FM (kg)] **Sarcopenia** assessed by DXA	Chest press, lat pulldown, knee extension, hamstrings curl, leg press, hip abduction, shoulder abduction, and orthostatic toe raises.	3	8–12	60–80%1RM	-	-	60	3 (-)	24	Yes
Vasconcelos et al. (28)	72 ± 4	F	BMI ≥30 kg/m^2^ and handgrip strength ≤ 21 kg **Sarcopenia** assessed by handgrip strength	Hip flexion with straight leg raise, Hip adduction, hip abduction, hip extension, hip flexion, knee flexion, knee extension, mini-squats with hips in a neutral position, and mini-squats with hips in external rotation.	2–3	8–12	40–75%1RM, 1–3 kg for hip exercises and mini-squats adjusted by the repetitions range	-	Low to “as fast as possible”	30-60	2 (85%)	10	Yes
Liao et al. (30)	66 ± 4	F	SMI < 7.15 kg/m^2^ and BF >30% **Sarcopenia** assessed by BIA	Seated chest press, seated row, seated shoulder press, concentric-eccentric hip circumduction, leg press, and leg curl	3	10–20	Elastic band color (load)and 13 RPE scale (maximum of 20)	-	Slowly performed	-	3 (-)	12	Yes
Park et al. (32)	73 ± 7	F	BMI ≥25 kg/m^2^ and ASM < 25.1% **Sarcopenia** assessed by BIA	Elbow flexion, wrist flexion, shoulder flexion, lateral raise, front raise, chest press, reverse flies, sideband, deadlift, squat, leg press, and ankle plantar flexion	2–3	8–15	13–17 RPE scale (maximum of 20)	-	-	60	3 (92%)	24	Yes
Huang et al. (29)	68 ± 4	F	SMI < 27.6% and BF >30% **Sarcopenia** assessed by BIA	1 or 2 types of exercise for shoulders, arms, lower limbs, chest, and abdomen	3	10	Elastic band color (load) and 13 RPE scale (maximum of 20)	-	Slowly performed	-	3 (-)	12	Yes
Chen et al. (33)	68 ± 4	F/M	M = ASM ≤ 32.5%; BMI ≥25 kg/m^2^ and VFA ≥100 cm F = ASM ≤ 25.7%; BMI ≥25 kg/m^2^ and VFA ≥100 cm **Sarcopenia** assessed by BIA	Shoulder presses, bicep curls, triceps curls, bench presses, deadlifts, leg swings, squats, standing rows, unilateral rows, and split front squats	3	8–12	60–70%1RM	-	-	120-180	2 (-)	8	Yes
	**Age**	**Sex**	**Sarcopenic obesity criteria**	**Exercises**	**Volume of sets**	**Repetitions**	**Load**	**Absolute intensity**	**Repetition cadence**	**Rest interval (s)**	**Weekly frequency (% of adherence)**	**Duration (weeks)**	**There was a progressive load during the study?**
Stoever et al. (35)	71 ± 4	F/M	M = SMI ≤ 37%; Grip strength ≤ 32 kg; BMI >28 kg/m^2^; SPPB score ≤ 8; gait speed 4 m < 0.8 m F = SMI ≤ 27.6%; Grip strength ≤ 21 kg; BMI >29 kg/m^2^; SPPB score ≤ 8; gait speed 4 m < 0.8 m **Sarcopenia** assessed by BIA	Knee extensors, elbow flexors and chest muscles, hip adductors and abductors, abdominal muscles, and back muscles	2–3	8–15	60–85%1RM	-	-	-	2 (-)	16	Yes
De Oliveira Silva et al. (38)	66 ± 3	F	The residual value (DXA measured AFFM - equation predicted AFFM) ≤ 3.4 BMI >27 kg/m^2^ **Sarcopenia** assessed by DXA	Chest press, 45° leg press, seated low row, leg extension, leg curl, triceps pulley extension, leg adduction, and abduction machines, standing arm curl, and seated calf raise	3	6–14	Adjusted by the repetitions range	Until concentric muscle failure	1-2s concentric 2s eccentric	-	2 (-)	16	Yes
Chiu et al. (34)	79 ± 7	F/M	M = SMM ≤ 37.15%; FM >29% F = SMM ≤ 32.26%; FM >40% **Sarcopenia** assessed by BIA	Arm curl, hand up, arm lateral raise, knee extension, calf raises, stepping, biceps curl, hand up, arm lateral raise and inversion, arm lateral raise and eversion, stepping, boxing, arms front, raise, arm adduction and abduction, hip flexion, foot dorsiflexion, and hand push	3	4–10	Adjusted by the repetitions range	-	-	30	2 (-)	12	Yes
	**Age**	**Sex**	**Sarcopenic obesity criteria**	**Exercises**	**Volume of sets**	**Repetitions**	**Load**	**Absolute intensity**	**Repetition cadence**	**Rest interval (s)**	**Weekly frequency (% of adherence)**	**Duration (weeks)**	**There was a progressive load during the study?**
Liao et al. (31)	66 ± 4	F	SMI < 27.6%; FM >30% **Sarcopenia** assessed by DXA	Seated chest press, seated row, seated shoulder press, knee extension, knee flexion, hip flexion, and hip extension	3	10-20	10-13 RPE scale (maximum of 20)	-	-	-	3 (-)	12	Yes
Nabuco et al. (37)	68 ± 4	F	ALST < 15.02 kg; FM ≥35% **Sarcopenia** assessed by DXA	Chest press, horizontal leg press, seated row, knee extension, preacher curl (free weights), leg curl, triceps pushdown, and seated calf raise	3	8–12	Adjusted by the repetitions range	-	-	-	3 (-)	12	Yes
Chang and Chiu (36)	81 ± 6	F/M	M = ASMI < 7 kg/m^2^; FM >29%; grip strength < 26 kg F = ASMI < 5.7 kg/m^2^; FM >40%; grip strength < 18 kg **Sarcopenia** assessed by BIA	Arm curl, hand up, arm lateral raise, knee extension, calf raises, stepping, biceps curl, hand up, arm lateral raise and inversion, arm lateral raise and eversion, stepping, boxing, arms front, raise, arm adduction and abduction, hip flexion, foot dorsiflexion, and hand push	3	4–10	Adjusted by the repetitions range	-	-	30	2 (65-73%)	12	Yes

F, female; M, male; SMI, skeletal muscle index; BMI, body mass index; BIA, bioelectrical impedance analysis; BF, body fat; ASM, appendicular skeletal muscle mass; FFM, fat-free mass; FM, fat mass; VFA, visceral fat area; SPPB, short physical performance battery; DXA, dual x-ray absorptiometry; AFFM, appendicular fat-free mass; SMM, skeletal muscle mass; ALST, appendicular lean soft tissue; ASMI, appendicular skeletal muscle index; 1RM, one repetition maximum; RPE, rating perceived exertion; -, no informed.

A scrutinized analysis of each variable of RT is presented below.

### 3.1. Resistance training protocol across studies

#### 3.1.1. Exercise selection

Regarding exercise selection, it is possible to observe a certain homogeneity in some selected exercises. For the multi-joint exercises for lower limbs, seven studies used the leg press (24, 25, 27, 28, 30, 32, 37, 38), four used squats (and their derivations) (26, 28, 32, 33), and two used deadlifts (32, 33). For the single-joint exercises for lower limbs, nine studies used knee extension (25, 27, 28, 30, 34–38), five used calf raise (24, 34, 36–38), five used hip flexion (27, 28, 30, 34, 36), four used hip abduction and adduction (24, 28, 35, 38), and two used knee flexion and hip extension (28, 30).

The multi-joint exercises for the upper limbs were composed of chest presses, lat pulldowns, and shoulder presses. In contrast, the single-joint exercises comprised biceps/triceps curls and shoulder abduction (24, 25, 27, 30–38). The studies of Shen et al. (26) and Huang et al. (29) did not detail the RT protocol and just provided information about the joints/muscles involved in each exercise prescribed.

#### 3.1.2. Volume of sets

A total of 10 studies fixed the number of sets (three sets) along the RT intervention (25, 26, 30–32, 34, 35, 37, 38), while five studies varied this number (one to three sets), as an alternative for load progression (27–29, 33, 35). Although the study by Shen et al. (26) proposed a training protocol, the researchers programmed an increase in training volume from more sets throughout the study (one to two sets in the initial protocol and two to three sets at the end).

#### 3.1.3. Repetitions

Overall, the number of repetitions ranged from 4 to 20. In four studies, it appears to range from 8 to 12 (25, 28, 33, 37) and in others, from 8 to 15 (27, 29) or 4 to 10 (34, 36). In two studies, the number of repetitions was fixed at 10 (27, 29).

#### 3.1.4. Load

Regarding the load, the studies varied in prescription methodology. A total of six studies prescribed the load by one repetition maximum, varying the percentage between 40 and 85% (24–26, 28, 33, 35). In total, five studies adjusted the weight by the repetition range (27, 34, 36–38), increasing the weight throughout the study, according to the progressive loading principles. In the studies that used the elastic band, the load was determined by the color/stretch of the band and the corresponding rating perceived exertion (i.e., with the change in the color of the elastic, the perception of effort changed) (29–32). Only one study reported RT intervention to absolute intensity (i.e., the endpoint of a set of repetitions) (38).

#### 3.1.5. Repetition cadence

Only five studies reported the repetition cadence as a variable (24, 28, 29, 31, 38). Across these studies, two were informed using numerical values (i.e., 1, 2, and 3 s). The study by Balachandran et al. (24) expressed the repetition cadence as “concentric as fast as possible and eccentric in 2 s,” and de Oliveira Silva et al. (38) used the concentric phase in 1–2 s and eccentric in 2 s. The other studies described it qualitatively. The studies by Liao et al. (30) and Huang et al. (29) reported the repetition cadence as “slowly performed”. The study by Vasconcelos et al. (28) used low and fast repetition cadences, progressing in the training protocol.

#### 3.1.6. Rest interval

A total of seven studies described the rest interval as a training variable, with values between 30 and 180 s (24, 25, 28, 32–34, and 36). In total, three of these studies described the rest interval in a defined range (e.g., 30–60, 60–120, and 120–180 s) (24, 28, 33). The other studies put forward a rest interval with a fixed value (between 30 and 60 s) (25, 32, 34, 36). A total of eight studies do not describe the rest interval between sets.

#### 3.1.7. Weekly frequency

All studies used between two and three exercise sessions per week. A total of seven studies used two sessions per week (24, 27, 28, 33–36), while the other seven stated three sessions (25, 26, 29–32, 37). Only four studies reported adherence to training frequency, ranging from 65 to 92% (24, 28, 32, 36).

## 4. Discussion

### 4.1. Main findings

This was the first study to examine the characteristics and main variables of RT protocols prescribed for older adults with SO. The review examined 15 studies and found that all of them reported the volume of sets, repetitions, load, and weekly frequency of the exercises. However, only 13 of the studies specified the exercise selection, and 10 studies did not detail the repetition cadence. Additionally, eight studies did not provide information on resting intervals between sets, indicating that not all studies have reported training protocols thoroughly. Therefore, the review highlights the need to improve the level of detail in exercise prescription for older adults with SO, as there are still gaps in the literature in this area.

### 4.2. The need for detailed descriptions of resistance training protocols

To ensure adequate total training volume and facilitate comparisons between studies, it is crucial to provide detailed and clear descriptions of RT variables that interact with mechanical stimuli and body adaptations in older adults (39). Furthermore, a detailed RT protocol description promotes its replication in clinical practice and comparisons of study results. Our review showed that many RT variables were relatively described, and the protocols were consistent with the current literature (15–17). However, some RT variables, such as repetition cadence and rest interval, were less well described.

#### 4.2.1. Repetition cadence

The repetition cadence is related to the concentric and eccentric phases of the movement, influencing the number of repetitions performed and the time under tension of each repetition (40). In our analysis of the studies, the repetition cadence was described in objective and subjective manners. Some studies reported the execution cadence in seconds, while others used terms such as “slowly” or “as fast as possible”. In cases of exercises performed “as fast as possible”, they can characterize a type of power training (41). The primary difference between RT and power training lies in the intensity of execution (42). RT employs heavier loads and longer execution times (39). Power training, in turn, requires lighter loads but with exercises performed faster (42). Consequently, including the term ‘as fast as possible' in an RT protocol may be a misunderstanding as it contradicts training principles (43). Despite this, many studies only describe their interventions as ‘RT,' which may not accurately reflect the nature of the training program employed (43).

Another point to be considered refers to the terms “repetition tempo” and “repetition cadence”. While tempo refers to the speed at which each repetition is performed (and consequently, the time under tension), the cadence is defined by a sequence of digits that correspond to particular movement phases (i.e., concentric and eccentric phases) (44). In this sense, the use of both terms can bring different interpretations in the analysis of the RT variables.

#### 4.2.2. Rest interval

Rest interval between sets is also an important variable because of its relation to the bioenergetics of the organic system and responses to repeated mechanical stimuli (45). Studies in the field of RT use this variable to compare strength and hypertrophy responses (46, 47). Longer intervals (more than 60 s) can allow for the maintenance of a higher total load lifted (48). Shorter intervals (<60 s) can decrease the total load lifted to make training denser (46). Moreover, the rest interval is associated with strength and hypertrophy outcomes, with a potential advantage for using long intervals. Therefore, the studies in RT could not fail to report the rest interval due to the possible interference of this variable on the intended outcome.

#### 4.2.3. The lack of clarification of the absolute intensity

The absolute intensity of the exercise is another point to be considered, as it refers to the endpoint at which the end of a set is established (49). The review proposed by Steele et al. (49) argues that the momentary muscle failure, repetitions in reserve, repetition maximum, and self-determined repetition maximum are ways to determine the end point of each set, as briefly described below. Momentary muscle failure happens when trainees are unable to complete the concentric portion of their current repetition, even with maximum effort. Repetitions in reserve refer to a pre-determined number of repetitions, despite the ability to complete additional repetitions (50). Repetition maximum, in turn, occurs when trainees complete the final possible repetition for which, if the next repetition was attempted, they would fail. The self-determined repetition maximum is another possibility for the endpoint of the sets, and it occurs when the trainee predicts a possible momentary failure on the following repetition (49). Although the studies report that the training prescription was determined by the %1RM or repetition range, they do not provide a clear scenario of the real endpoint of the sets, as demonstrated in our study (Table 1). If the absolute intensity aspects that may influence the total load lifted and the periodization program, along with the intervention, are not clarified, it could be considered a misconception in the RT programming strategy (51, 52). Reporting the endpoint of the sets in more detail in the studies can fill this gap of the absolute training intensity.

#### 4.2.4. The exercise order is not clear

In our selected studies, we identified another gap related to the order in which exercises were performed. The order of execution seems to have some chances of dependence syndrome. Even though different exercise orders did not affect chronic adaptations in muscle strength, hypertrophy, anabolic hormones, magnitude, and duration of postexercise hypotension in healthy older women (53, 54), it was not possible to assume the same responses in older adults with SO. Indeed, older adults with SO potentially present impaired physical performance (55), muscle strength, and quality (4), which together can be responsible for triggering different exercise responses according to exercise order (56). Therefore, a critical evaluation of exercise order is recommended when dealing with older adults with SO, since training performance can be compromised throughout the session. Although there is still a lack of sufficient evidence regarding the impact that the exercise order exerts on physiological outcomes, the absence of this information in the study methods represents a constraint of it.

### 4.3. Exercise selection cannot be a forgotten variable

One concern raised in our review was the insufficient description of exercise selection in two studies. The selection of exercise that is most suitable for achieving training objectives and addressing the specific needs of the target audience should be among the primary concerns of strength and conditioning coaches. This indication failed in particular in 2 of the 15 studies analyzed (24–38), especially considering the specificity of the population of older adults with SO. During aging, the most significant reduction in muscle strength in the lower limbs occurs in the quadriceps with values reaching up to 76% in knee extensors (57, 58). Increasing the work for the quadriceps muscle can attenuate the muscle and function losses. In addition, helps to maintain the balance of forces on other thigh muscles (i.e., hamstrings) (59, 60). Imaging analysis studies throughout aging show a stabilization of the muscle volume of the hamstrings, concomitantly with a reduction in the muscle volume of the quadriceps (61), which reinforces the need for resistance exercises to strengthen the anterior region of the thigh. In this sense, the leg press may be a suitable exercise for older adults because of the minimal load placed on the spine compared to squats with an axial load (62).

The selection of appropriate exercises in RT is crucial for understanding the specific muscle groups targeted by each exercise (62). A rationale exercise prescription should begin with a consideration of the anatomical and biomechanical principles to understand the contractile behavior (63) of the muscle as a function of the workload imposed (64). It is important to note that the differences between exercises performed using machines, free weights, and elastic bands can significantly impact the muscle worked, the torques applied, and the angles involved in different phases of movement (65). Most of the studies in our review used knee extension and leg press exercises to target the lower limbs. The muscles worked during knee extension (especially the rectus femoris) and leg press (total quadriceps femoris and gluteus) have an essential role in the physical performance of older adults (66, 67). However, the execution of different types of equipment or instruments can affect the training intensities (65). These differences in training protocols make it difficult to compare the findings of the studies, as well as their replication in different conditions/equipment models (68). Even so, regardless of the eventually divergent results for the lower limbs, this is one of the most sensitive segments of older adults (69, 70). Therefore, it matters that they are worked on with rationally well-selected exercises.

### 4.4. Recommendations for detailing each variable

**Figure 2:**
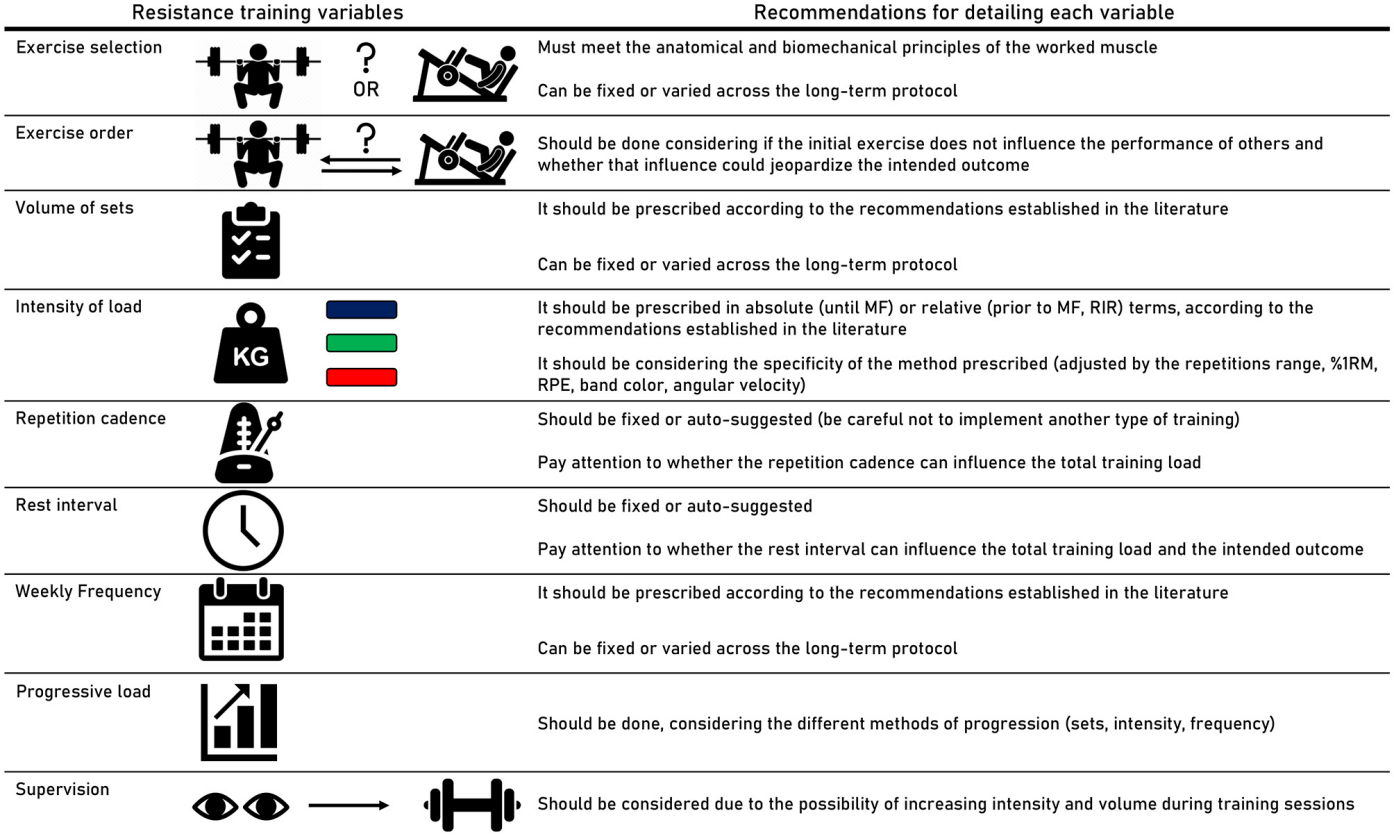
Recommendations for detailing the characteristics of resistance training protocols prescribed for older adults. MF, momentary failure; RIR, repetitions in reserve; 1RM, one repetition maximum; RPE, rating perceived exertion.

We provide expected easy-to-understand recommendations (Figure 2) for each RT variable used in prescribing RT for older adults with SO, whether in application in research or clinical practice. We consider the eight main dimensions as follows: “exercise selection,” “exercise order,” “volume of sets,” “intensity of load,” “repetition cadence,” “rest interval between sets,” “weekly frequency,” and “progressive load”. In addition, we consider that RT under specialized supervision may favor the use of exercise with greater efforts and greater safety for older adults (71). The recommendations were created considering the current literature in the field of RT (49, 64, 72, 73). From an evidence-based approach (74), it is relevant that studies on RT for older adults with SO consider greater detail in the training protocol.

### 4.5. Limitations of the study

#### 4.5.1. Studies included

Some limitations of our review need to be considered. The first lies in the small number of included studies, despite its initial number (1,693). The relatively rigorous inclusion and exclusion criteria may have contributed to this, although narrowing the research question of our review was necessary to gain a better understanding of how studies are detailing the RT protocol for older adults with SO. Furthermore, the natural scarcity of studies involving this specific population of older adults is another factor that may have contributed to this limitation.

#### 4.5.2. Absence of outcome analysis

Another limitation involves the absence of outcome analysis, which may confuse the interpretation of the studies. For example, we neither considered the effects of RT on increasing muscle mass or physical performance in older adults nor did we conduct an interpretation of an eventual improvement in some outcomes in older adults with SO. To report the expected outcomes, we would need to conduct a risk of bias analysis (75) and weigh the experimental designs of the studies (randomized controlled trials, not randomized, or not controlled) (19). The scope of our review was focused on analyzing the RT protocols so that future studies conduct their experiments more carefully, without the intervention being able to change a secondary outcome (21).

#### 4.5.3. Methods used to determine SO

Another point that deserves caution involves the evaluation of the methods used to determine SO. We included studies with a diagnosis of SO according to criteria well established in the literature (11). There is still no consensus on which are the best and most appropriate measurement methods (sarcopenia by DXA or BIA; obesity by BMI or bodyfat%) of the components that involve the SO (11, 76). These discussions mainly involve the criterion for determining sarcopenia, which can vary widely. It is worth noting that the fat-free mass corrected for fat-free adipose tissue (estimated from DXA measurements) should be considered since the latter may have a potential confounding effect when assessing the prevalence of sarcopenia (77). Therefore, reaching a consensus on the definition of sarcopenia is crucial to advance research in the field, and more importantly, to determine the prognostic value of a sarcopenia diagnosis and the appropriate RT strategies for affected older adults (78).

However, as our study did not perform the outcome analysis, the different measurement methods do not impact our findings. Nevertheless, we suggest that the evaluation of SO should be considered in future studies to avoid biases in the misclassification of the syndrome (79).

#### 4.5.4. Other types of resistance training

Our study only considered RT protocols that used free weights, machines, or elastic bands. However, the literature presents other types of RT that can be considered, such as RT in suspension or on unstable platforms. These types of RT require a certain level of balance and coordination, which may be challenging for older adults with SO. This could increase the risk of falls or injury if proper form and technique are not maintained (80). Moreover, suspension and unstable platform exercises may not be suitable for individuals with existing injuries or conditions that affect their joints or balance, as the instability of the equipment could exacerbate these issues (81).

### 4.6. Practical application and future research directions

#### 4.6.1. Implications for clinical practice

In terms of clinical practice, strength and conditioning coaches who prescribe RT for older adults with SO should take our evidence mapping into account. Both free weights and machines can be used for the upper and lower limbs, and the variables can be rationalized for better adaptation to the training scenario. The prescription of volume, load, repetition time, rest interval, and weekly frequency should be reported and planned to enable trainers to manipulate these variables effectively. The selection and order of the exercises should be thoughtfully considered to ensure adequate load progression throughout the training. Moreover, our recommendations carefully outline what each variable should contain when prescribing RT for older adults (see Figure 2).

#### 4.6.2. Prospects for future studies

Future research should focus on providing detailed prescriptions of RT for older adults with SO. Comparisons of RT protocols across different conditions, such as sarcopenia, obesity, and SO, may help determine if prescribing can be standardized or personalized based on the condition. Another suggestion is to explore the impact of RT variables (e.g., the volume of sets), verifying whether they can be decisive for body recomposition and the attenuation of the clinical condition of older adults with SO. Mapping training prescriptions for other aging conditions, such as only sarcopenia, can be a prospect for future studies to ensure adequate detailing of training planning and its variables. Moreover, future studies should consider implementing the Proper Reporting of Evidence in Sport and Exercise Nutrition Trials (PRESENT) guidelines to address specific aspects of the combination of sports nutrition and exercise metabolism fields (82).

## 5. Conclusion

The characteristics of RT prescribed for older adults with SO were mapped, understanding the combination between the RT variables and forms of intervention among the studies. Surprisingly, we found some gaps that are not common in the field of RT. The lack of description regarding specific exercise selection, repetition cadence, and resting interval are critical points that our study reported. The proposed easy-to-understand recommendations may contribute to a reflection in the clinical practice and research regarding greater training control and RT variables of older adults with SO.

## Author contributions

LS and LG conducted experiments and wrote the introduction, methods, results, and discussion sections. PA, CB, MT, AV, LB, and PM-P improved the interpretation analysis and reviewed the manuscript. JM and WK drafted the manuscript, improved the interpretation analysis, and reviewed English grammar and spelling. AS and DM supervised the study, drafted the manuscript, and gave final approval for the version submitted for publication. All authors contributed to the article and approved the submitted version.
