# Supplementary material for: Characteristics of resistance training-based protocols in older adults with sarcopenic obesity: a scoping review of training procedure recommendations
Source: Front Nutr. 2023 May 10;10:1179832. doi: 10.3389/fnut.2023.1179832 (PMC10206023; doi:10.3389/fnut.2023.1179832)
Supplement: Supplementary file 2 [file Table_2.docx]

Supplementary Table 2. Detailed search strategy in each specific base.

| **Database** | **Search strategies** |
| --- | --- |
| PubMed | (("resistance training" or "resistance exercise" or "strength training" or "strength exercise" or "weight exercise" or "weight training" or “exercise training”) AND (sarcopeni*) AND (obes*)) |
| Web of Science | Same of PubMed |
| EMBASE | Same of PubMed |
| LILACS | Same of PubMed |
| Cochrane Library | Same of PubMed |
| SCOPUS | Same of PubMed |
| Google Scholar* | ("resistance training" OR "resistance exercise") AND (sarcopenia OR sarcopenic) AND (obesity OR obese) |
| Medrxiv | Same of Google Scholar |
